# Supplementary material for: Phagocytosis of Plasmodium falciparum ring-stage parasites predicts protection against malaria
Source: Nat Commun. 2022 Jul 14;13:4098. doi: 10.1038/s41467-022-31640-6 (PMC9281573; doi:10.1038/s41467-022-31640-6)
Supplement: Supplementary file 1 — Supplementary Information [file 41467_2022_31640_MOESM1_ESM.pdf]

Supplementary Data

Article title: Phagocytosis of *Plasmodium falciparum* ring-stage parasites predicts protection against malaria

Supplementary Table 1: *Plasmodium* proteins identified by mass spectrometry

No.	PlasmoDB identifier	Product description	Molecular weight (kDa)	Protein length	Signal peptide	Trans-membrane Helix prediction	Predicted function
1	PF3D7_1324900	Lactate dehydrogenase	34.107	316	yes	yes	carbohydrate metabolic process
2	*PF3D7_0929400	High molecular weight rhoptry protein 2 (RhopH2)	162.663	1378	yes	no	entry into host cell
3	*PF3D7_0905400	High molecular weight rhoptry protein 3 (RhopH3)	104.854	897	yes	no	entry into host cell, host cell surface receptor binding
4	*PF3D7_0731500	Erythrocyte binding antigen 175 (EBA-175)	174.586	1502	yes	yes	entry into host cell; host cell surface receptor binding
5	*PF3D7_1301600	Erythrocyte binding antigen 140 (EBA-140)	140.594	1210	yes	yes	host cell surface receptor binding
6	*ENO	Enolase	50	446			entry into host cell
7	PF3D7_0818900	Heat shock protein 70;70kDa	73.914	677	no	no	response to stress; ATPase activity
8	PCYB_112350	Elongation factor 1	48.011	434	no	no	translational elongation
9	PF3D7_0917900	Heat shock protein 70;78kDa	72.386	652	yes	no	response to drug
10	PKH_093790	Myosin heavy chain subunit	199.670	1718	no	no	Myosin complex
11	PY07151	Uncharacterized protein	81.098	690	no	no	no data available
12	PF3D7_1027300	Peroxiredoxin	43.929	393	no	no	entry into host cell & antioxidant activity
13	*PF3D7_0302500	Cytoadherence linked asexual protein (RhopH1/Clag3.1)	167.240	1417	yes	no	cytoadherence to microvasculature, mediated by symbiont protein
14	*PF3D7_1410400	Rhoptry-associated protein 1 (RAP1)	90.052	782	yes	no	protein binding
15	*PF3D7_0501600	Rhoptry-associated protein 2 (RAP2)	46.738	398	yes	no	entry into host cell; protein binding
16	PF3D7_0626800	Pyruvate kinase	55.660	511	no	no	carbohydrate metabolic process
17	PF3D7_0216400	Vacuolar protein sorting associated protein 45	86.255	722	no	no	vesicle docking involved in exocytosis

18	PF3D7_1311800	M1-family alanyl aminopeptidase	126.061	1085	no	yes	Proteolysis; response to drug
19	PF3D7_1029600	Adenosine deaminase	42.465	367	no	no	purine ribonucleoside monophosphate biosynthetic process; deaminase activity
20	PF3D7_1012400	Hypoxanthine-guanine phosphoribosyl Transferase	26.362	231	no	no	purine ribonucleoside salvage; hypoxanthine phosphoribosyl transferase activity
21	PF3D7_1451100	Elongation factor 2	93.521	832	no	no	translational elongation
22	PF3D7_1406500	WD repeat-containing protein 65	181.532	1527	no	no	cytoskeletal protein binding
23	PF14_0425	Fructose-bisphosphate aldolase	40.104	369	no	no	carbohydrate metabolic process; cytoskeleton organisation
24	PF3D7_1462800	Glyceraldehyde 3-phosphate dehydrogenase	36.635	337	no	no	carbohydrate metabolic process
25	PVX_089505	Suppressor of Ras1 3-9	30.220	262	no	no	protein domain specific binding
26	PVX_123890	Uncharacterized protein	137.424	1265	no	no	chromatin binding; DNA binding
27	PVX_081792	Uncharacterized protein	576.792	5031	no	no	microtubule-based movement; ATPase activity
28	C922_03767	CTP synthase	95.801	850	no	no	pyrimidine nucleotide biosynthetic process
29	C922_03524	Uncharacterized protein	398.898	3500	no	no	Protein binding
30	PCYB_147060	Actin	39.931	356	no	no	no data available
31	PCYB_142080	ATP synthase subunit gamma	31.331	274	no	no	ATP synthesis coupled proton transport
32	PCYB_053170	Uncharacterized protein	660.712	5758	no	no	no data available
33	PY02078	Uncharacterized protein	79.631	674	no	yes	no data available
34	PBANKA_134200	SNARE protein	44.948	416	no	yes	no data available

Numbers 1-7: proteins that were differentially identified between experimental conditions. Numbers 8-11**:** proteins that were comparably identified between experimental conditions. Numbers 12-34: proteins that were identified but did not meet the stringent statistical criteria for analysis. The proteins were categorized based on Uniprot. * Merozoite proteins associated with rhoptries, micronemes or the merozoite

Supplementary Table 2: Number of unique peptides & label free quantification values (LFQ) for n= 34 *Plasmodium* proteins identified by mass spectrometry

No.	Plasmodb identifier	Product description	Razor + unique peptides				Label Free Quantification (LFQ) Log2 values			
			uEs_used	uEs_fresh	Ring culture _shaved	Ring culture _mock shaved	uEs_used	uEs_fresh	Ring culture _shaved	Ring culture _mock shaved
1	PF3D7_1324900	Lactate dehydrogenase	0,3,3,3	0,0,0,0	3,3,3,3	0,1,1,0	0,22.9, 23.9,24.3	0,0,0,0	23.8,23.5, 24.1,25.3	0,0,0,0
2	*PF3D7_0929400	High molecular weight rhoptry protein 2 (RhopH2)	1,3,0,0	0,0,0,0	4,3,2,8	1,0,0,1	0,0,0,0	0,0,0,0	25,23.7, 23.8,24.2	0,0,0,0
3	*PF3D7_0905400	High molecular weight rhoptry protein 3 (RhopH3)	0,2,0,1	0,0,0,0	4,3,2,5	2,1,1,0	0,21.7,0,0	0,0,0,0	24.2,22.4, 22.7,23.4	23.9,0,0,0
4	*PF3D7_0731500	Erythrocyte binding antigen 175 (EBA-175)	9,15,10,9	0,0,0,0	4,9,7,10	3,5,4,5	26.8,26.3, 26.3,25.9	0,0,0,0	22.5,25, 23.8,25.3	23.1,23.6, 24,24.9
5	*PF3D7_1301600	Erythrocyte binding antigen 140 (EBA-140)	8,11,7,7	0,0,0,0	3,9,6,12	1,1,0,1	26.1,26.3, 26.1,26.4	0,0,0,0	24.4,25.6, 25.4,26.1	0,0,0,0
6	*ENO	Enolase	0,0,0,0	0,0,0,0	3,5,3,5	0,0,0,0	0,0,0,0	0,0,0,0	23,22.8, 22.6,24.8	0,0,0,0
7	PF3D7_0818900	Heat shock protein 70;70kDa	0,0,0,0	0,0,0,0	3,0,2,3	0,1,0,0	0,0,0,0	0,0,0,0	23.6,0, 23.7,24	0,0,0,0
8	PCYB_112350	Elongation factor 1	1,2,1,1,	0,0,0,0	1,2,2,4	0,0,1,1	0,23.8,0,0	0,0,0,0	0,24, 24.7,25.1	0,0,0,0
9	PF3D7_0917900	Heat shock protein 70;78kDa	3,3,2,2	3,2,3,3	5,6,6,9	3,4,4,2	27.8,27.7, 27.1,27	27.8,27, 27.7,27.3	28.3,28.1, 27.7,27	28.7,28.6, 28.4,27.4
10	PKH_093790	Myosin heavy chain subunit	1,1,1,1	1,1,1,1	1,1,1,1	1,1,1,1	25.6,25, 25.8,26	26,0, 26.3,0	25.4,25.1, 26,25.5	26.1,26.3, 26.2,25.6
11	PY07151	Uncharacterized protein	1,1,1,1	1,1,1,1	1,1,1,1	1,1,1,1	28.6,28.4, 0,28.6	0,28.7, 29,0	29,0, 0,28.7	0,0, 0,28.8

12	PF3D7_1027300	Peroxiredoxin	0,0,0,0	0,0,0,0	0,2,2,4	0,0,0,0	0,0,0,0	0,0,0,0	0,20.9, 0,23	0,0,0,0
13	*PF3D7_0302500	Cytoadherence linked asexual protein (RhopH1/Clag3.1)	0,0,0,0	0,0,0,0	3,1,1,1	1,0,0,0	0,0,0,0	0,0,0,0	24.5,0, 0,0	0,0,0,0
14	*PF3D7_1410400	Rhoptry-associated protein 1 (RAP1)	0,1,0,0	0,0,0,0	0,1,0,2	0,0,0,0	0,0,0,0	0,0,0,0	0,0, 0,21.5	0,0,0,0
15	*PF3D7_0501600	Rhoptry-associated protein 2 (RAP2)	1,1,1,1	0,0,0,0	0,1,1,2	0,0,0,0	0,0,0,0	0,0,0,0	0,0, 0,22.7	0,0,0,0
16	PF3D7_0626800	Pyruvate kinase	0,0,0,0	0,0,0,0	0,0,0,2	0,0,0,0	0,0,0,0	0,0,0,0	0,0, 0,21.6	0,0,0,0
17	PF3D7_0216400	Vacuolar protein sorting-associated protein 45	1,1,1,1	1,1,1,1	1,1,1,1	1,1,1,1	0,0,0,0	0,0,0,0	0,0,0,0	0,0, 0,26.8
18	PF3D7_1311800	M1-family alanyl aminopeptidase	0,0,0,0	0,0,0,0	0,0,0,1	0,0,0,0	0,0,0,0	0,0,0,0	0,0, 0,20.8	0,0,0,0
19	PF3D7_1029600	Adenosine deaminase	0,0,0,0	0,0,0,0	0,1,1,1	0,0,0,0	0,0,0,0	0,0,0,0	0,0,0,21	0,0,0,0
20	PF3D7_1012400	Hypoxanthine-guanine phosphoribosyl Transferase	0,0,0,0	0,0,0,0	0,0,0,2	0,0,0,0	0,0,0,0	0,0,0,0	0,0, 0,21.7	0,0,0,0
21	PF3D7_1451100	Elongation factor 2	1,1,2,1	0,0,0,0	0,0,0,2	0,1,1,0	0,0,0,0	0,0,0,0	0,0, 0,22.1	0,0,0,0
22	PF3D7_1406500	WD repeat-containing protein 65	1,1,1,1	1,1,1,1	1,1,1,1	1,1,1,1	0,0,0,0	0,0,0,0	0,0,0,0	0,0, 0,26.8
23	PF14_0425	Fructose-bisphosphate aldolase	0,0,0,0	0,0,0,0	0,0,0,1	0,0,0,0	0,0,0,0	0,0,0,0	0,0, 0,22.5	0,0,0,0
24	PF3D7_1462800	Glyceraldehyde 3-phosphate dehydrogenase	1,2,2,1	1,0,1,1	1,1,2,4	0,1,0,0	0,0,0,0	0,0,0,0	0,0, 27.6,0	0,0,0,0
25	PVX_089505	Suppressor of Ras1 3-9	0,0,0,0	0,0,0,0	0,0,0,1	0,0,0,0	0,0,0,0	0,0,0,0	0,0, 0,21.5	0,0,0,0
26	PVX_123890	Uncharacterized protein	1,0,1,0	1,1,1,1	1,0,1,0	0,1,1,1	0,0,0,0	0,0,0,0	0,0,0,0	0,0,0,23
27	PVX_081792	Uncharacterized protein	0,0,0,0	0,0,1,1	0,1,0,0	0,0,1,1	0,0,0,0	0,0,0,0	0,0,0,0	0,0,0,0
28	C922_03767	CTP synthase	1,1,1,1	1,1,1,1	1,1,1,1	1,1,1,1	0,0,0,0	0,0,0,0	0,0,0,0	0,0, 0,24.3

29	C922_03524	Uncharacterized protein	1,0,0,1	0,0,0,0	1,0,0,0	0,0,0,0	0,0,0,0	0,0,0,0	0,0,0,0	0,0,0,0
30	PCYB_147060	Actin	0,1,0,1	0,0,0,0	0,1,0,0,	0,0,0,0	0,0,0,0	0,0,0,0	0,0, 0,22.3	0,0,0,0
31	PCYB_142080	ATP synthase subunit gamma	1,0,0,1	0,0,0,0	1,1,0,0	0,1,1,0	0,0,0,0	0,0,0,0	0,0,0,0	0,0,23.0,0
32	PCYB_053170	Uncharacterized protein	0,0,1,1	0,0,0,0	0,0,0,0	0,0,0,1	0,0,0,0	0,0,0,0	0,0,0,0	0,0,0,22.5
33	PY02078	Uncharacterized protein	0,0,1,1	1,1,1,1	0,0,1,1	0,1,1,0	0,0,0,0	0,0,0,0	0,0,0,0	0,0,24.3,0
34	PBANKA_134200	SNARE protein	0,0,0,0	0,0,0,0	1,1,0,0	0,1,1,1	0,0,0,0	0,0,0,0	0,0,0,0	0,0,0,27.9

Numbers 1-7: proteins that were differentially identified between experimental conditions. Numbers 8-11**:** proteins that were comparably identified between experimental conditions. Numbers 12-34: proteins that were identified but did not meet the stringent statistical criteria for analysis. To pass the selection criteria, proteins had to have: i) a minimum of 2 unique peptides in order to get a LFQ value, and ii) LFQ values > 0 in three of four independent replicates for at least one experimental condition. *Merozoite proteins associated with rhoptries, micronemes or merozoite surface.

Supplementary figures

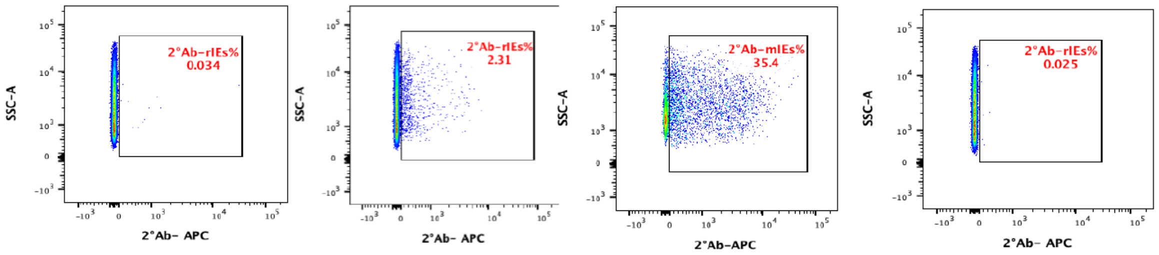

Supplementary Fig. 1 Detection of antibody binding to ring-infected erythrocytes (rIEs) by flow cytometry Malaria-immune plasma also show modest levels of binding to rIEs in comparison to mIEs using flow cytometry. Negligible binding detected with malaria-naïve serum or with secondary antibody in the absence of malaria-immune plasma. A minimum of 100,000 cells were acquired for each experimental setup. Experiments were conducted at least 20 times as these samples were used as controls and thus included in many subsequent experiments. Similar results were obtained each time. Each blue dot represents a single cell. Source data are provided as a Source Data file.

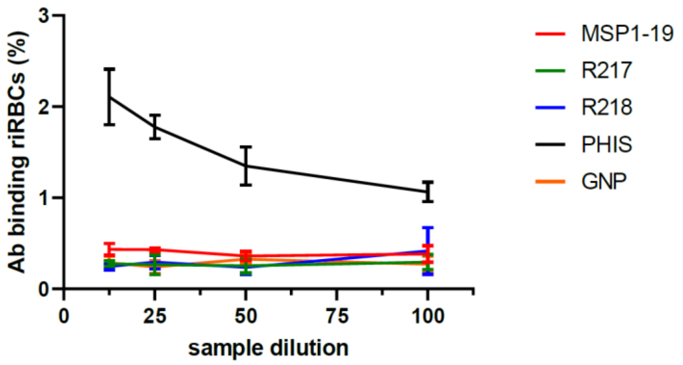

Supplementary Fig. 2 Antibody binding to ring-infected erythrocytes (rIEs). Monoclonal antibodies (mAbs) R217 (green line) and R218 (blue line) against EBA-175 do not bind to rIEs. Negative controls included a mouse mAb against the 19 Kilodalton fragment of merozoite surface protein 1 ($MSP1_{19}$, red line) and a pool malaria-naïve plasma from donors in Germany (GNP, German naïve plasma, orange line). The positive control was PHIS; pooled human immune sera from Kenyan adults, black line. Error bars show the mean and standard error of the mean (SEM). A total of n = 5 biologically independent samples were analyzed in serial dilutions in duplicate in n = 2 independent experiments. Source data are provided as a Source Data file.

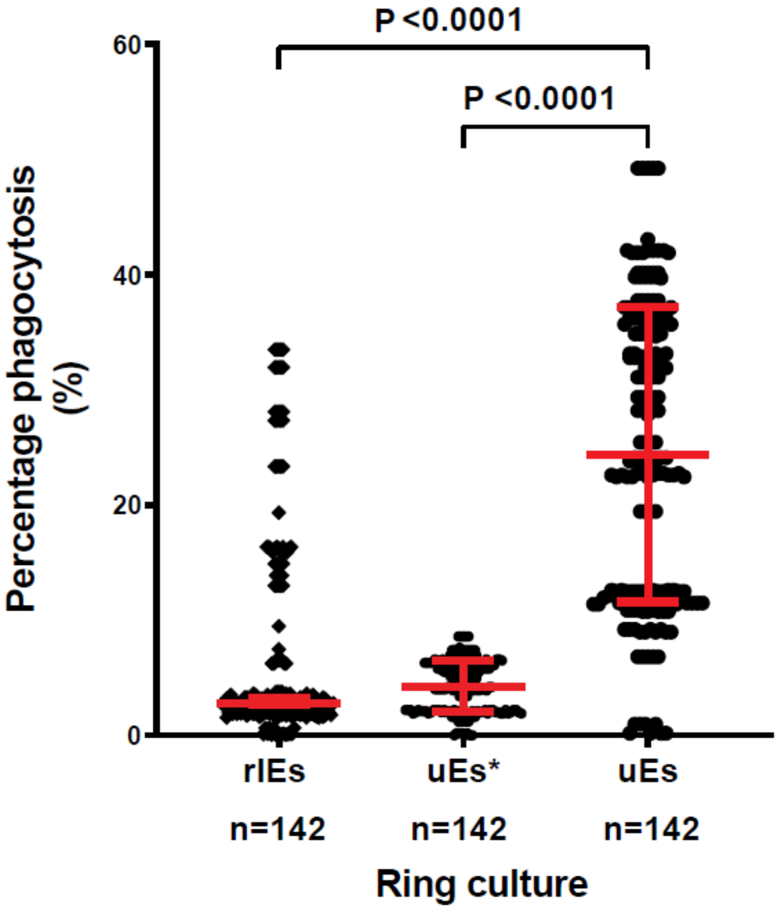

Supplementary Fig 3: Phagocytosis of uninfected erythrocytes (uEs) was higher than that of ring-infected erythrocytes (rIEs). The proportion of phagocytosed rIEs (closed diamonds), was compared to that of uEs (open diamonds), and to the proportion of uEs normalized (uEs*, open circles) to the ring culture parasitaemia. Each data point represents the RPI measured for one plasma sample with 2 technical replicates. A total of N = 142 biologically independent samples were analyzed for rIEs, uEs and normalized uEs. Red lines show the median and the 95% confidence intervals. P values were determined by the Kruskal-Wallis two-sided test followed by Dunns's multiple comparison test. Source data are provided as a Source Data file.

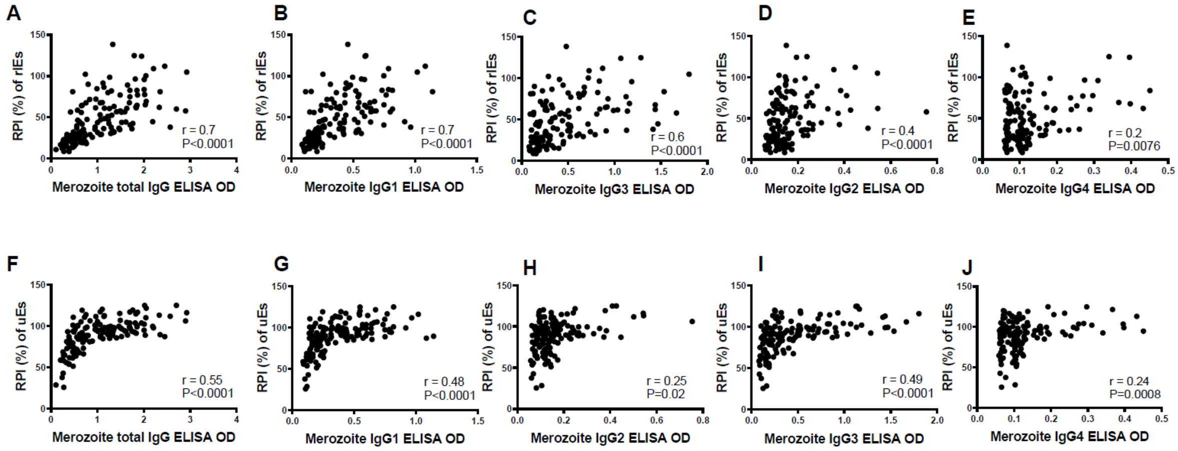

Supplementary Fig. 4: Phagocytosis of ring-infected and uninfected erythrocytes is strongly correlated with antibodies against merozoites. Correlations between the relative phagocytosis index (RPI) of rIEs (A-E) and uEs (F-J) and the presence of antibodies against merozoites (shown as ELISA OD values). Correlations with total IgG (A and F), cytophilic subclasses IgG1 and IgG3 (B, C and G,H), and non-cytophilic subclasses IgG2 and IgG4 (D,E and I,J). Data points in each panel are from N = 142 biologically independent samples analysed in duplicate in a single experiment. Pair-wise correlations were analysed using Spearman’s R with accompanying P values. Source data are provided as a Source Data file.

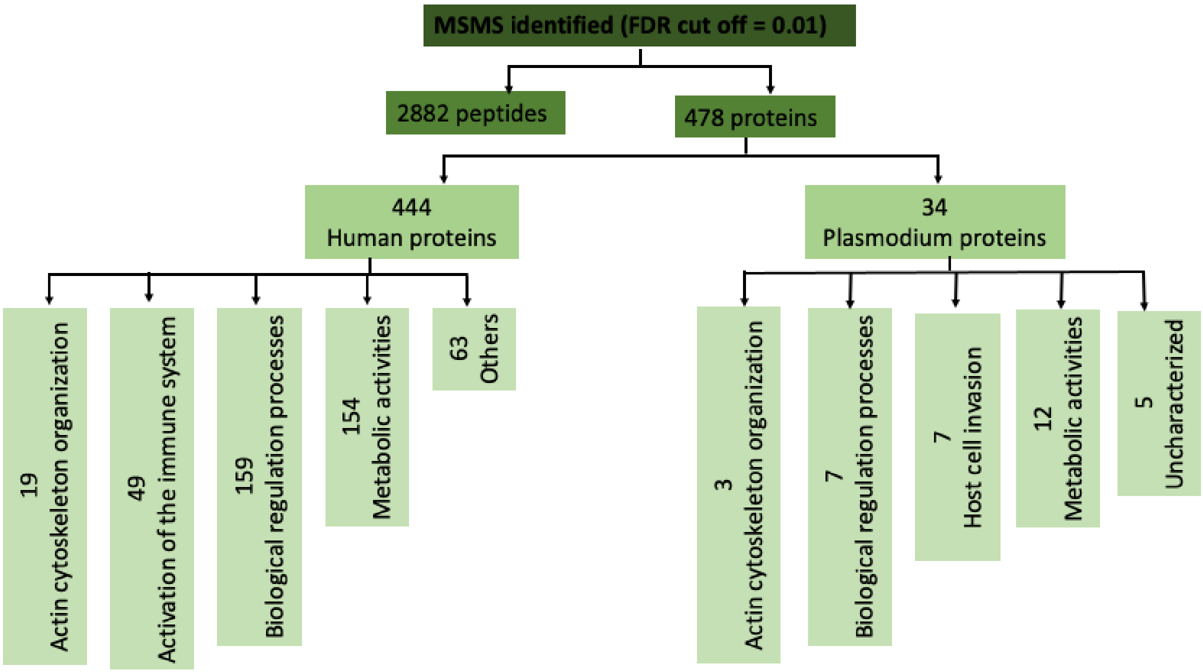

Supplementary Fig. 5 Categorization of the identified proteins. Among the 478 identified peptides, there were 444 human proteins and 34 *Plasmodium* proteins. The human proteins were categorised based on the gene ontology of biological processes; 19 - for actin cytoskeleton organization, 49 - for activation of the immune system, 159 - for biological regulation processes, 154 - for metabolic activities and 63 - for other cellular biological processes. The *Plasmodium* proteins were also categorised based on the gene ontology of biological processes 3 - for actin cytoskeleton organization, 7 - for biological regulation processes, 12 - for metabolic activities, 7 - for host cell invasion and 5 - were uncharacterized. The categorization was based on Uniprot. Source Data are uploaded onto PRIDE.

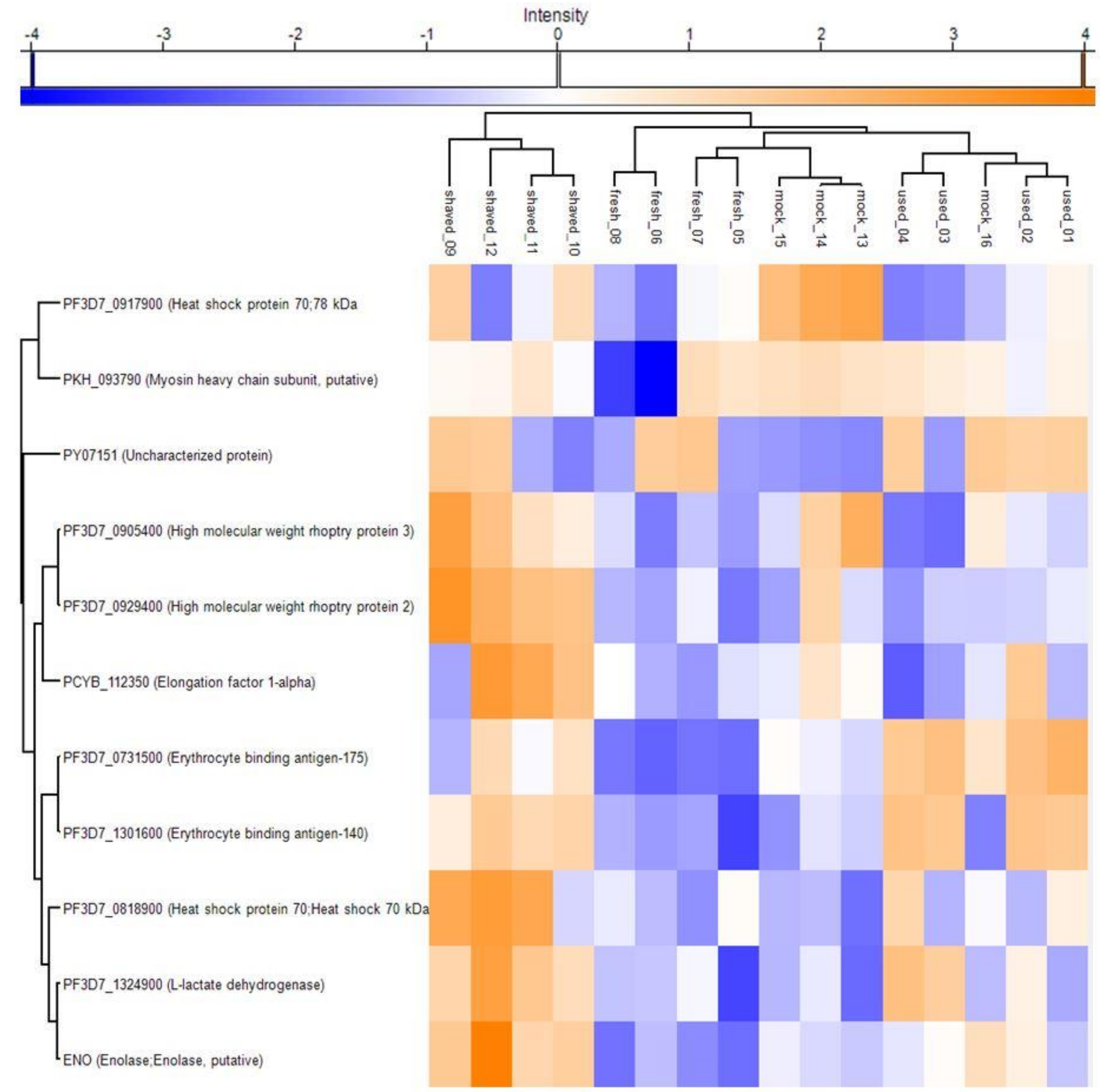

Supplementary Fig. 6 Heatmap generated using only the selected *Plasmodium* proteins. The heatmap was generated using Log-2 LFQ values that had been Z-score normalized and hierarchical clustering was performed on row and columns using the Euclidean distance and ward.D2. Quantitative values have been colour coded according to the colour key at the top of the figure. Proteins are labelled by their gene-name and accession number. Sample names are shown on the top of the plot. The samples were supernatant obtained after trypsin-shaving cells from different cultures: used_01-04 from uEs in used media; fresh_05-08 from uEs in fresh media; shaved_09-12 from trypsin-shaved ring culture and mock_13-16 from mock-shaved ring culture. The proteins used to generate the heatmap were those that passed the selection criterion for statistical analysis. Source Data are uploaded onto PRIDE.

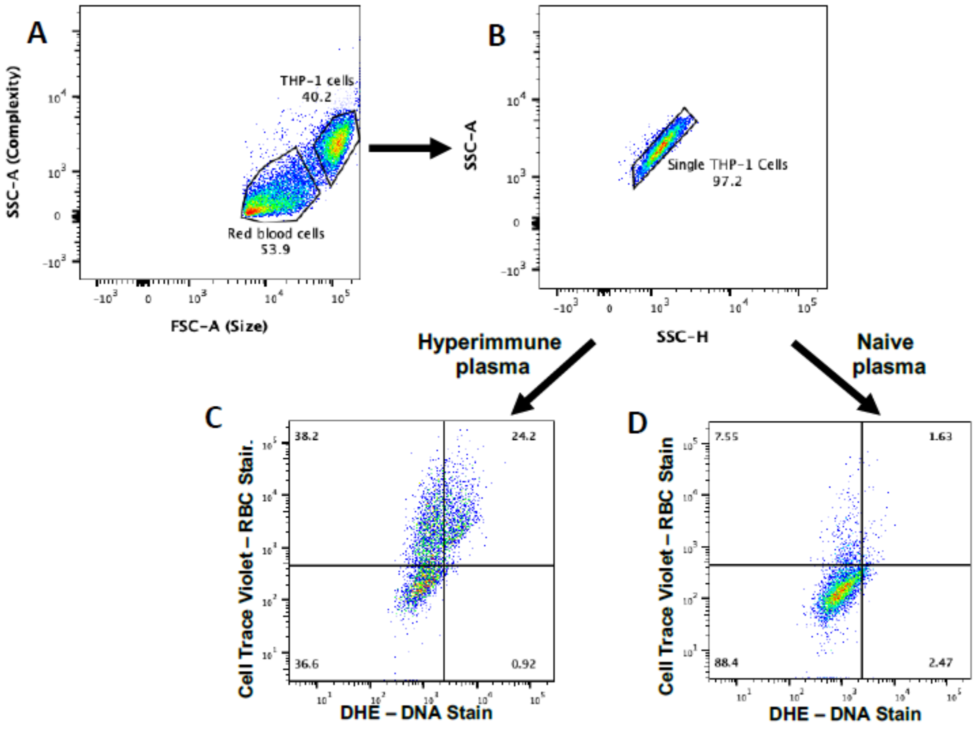

Supplementary Fig. 7 Flow cytometry gating strategy for opsonic phagocytosis of ring-infected erythrocytes. A) The photomultiplier tube PMT voltages of the forward scatter and side scatter were adjusted to ensure that the population of THP-1 cells and red blood cells were well discriminated based on their difference in size and complexity. B) Only the single cells of our population of interest (THP-1 cells) that passed the laser beam in the flow cell were gated. The PMT voltage of CellTrace Violet and DHE dyes were adjusted using relevant controls in order to identify and correctly discriminate the true population of THP-1 cells that phagocytosed uEs (top right quadrant) from those that phagocytosed rIEs (top left quadrant) after opsonization with (C) hyperimmune and (D) naïve plasma. Source data are provided as a Source Data file.
