# Supplementary material for: Phagocytosis of Plasmodium falciparum ring-stage parasites predicts protection against malaria
Source: Nat Commun. 2022 Jul 14;13:4098. doi: 10.1038/s41467-022-31640-6 (PMC9281573; doi:10.1038/s41467-022-31640-6)
Supplement: Supplementary file 3 — Reporting Summary [file 41467_2022_31640_MOESM3_ESM.pdf]

nature portfolio

Corresponding author(s): Faith Osier

Last updated by author(s): May 28, 2022

Reporting Summary

Nature Portfolio wishes to improve the reproducibility of the work that we publish. This form provides structure for consistency and transparency in reporting. For further information on Nature Portfolio policies, see our Editorial Policies and the Editorial Policy Checklist.

Statistics

For all statistical analyses, confirm that the following items are present in the figure legend, table legend, main text, or Methods section.

n/a	Confirmed	
☐	☒	The exact sample size (n) for each experimental group/condition, given as a discrete number and unit of measurement
☐	☒	A statement on whether measurements were taken from distinct samples or whether the same sample was measured repeatedly
☐	☒	The statistical test(s) used AND whether they are one- or two-sided<br>*Only common tests should be described solely by name; describe more complex techniques in the Methods section.*
☐	☒	A description of all covariates tested
☐	☒	A description of any assumptions or corrections, such as tests of normality and adjustment for multiple comparisons
☐	☒	A full description of the statistical parameters including central tendency (e.g. means) or other basic estimates (e.g. regression coefficient) AND variation (e.g. standard deviation) or associated estimates of uncertainty (e.g. confidence intervals)
☐	☒	For null hypothesis testing, the test statistic (e.g. F, t, r) with confidence intervals, effect sizes, degrees of freedom and P value noted<br>*Give P values as exact values whenever suitable.*
☒	☐	For Bayesian analysis, information on the choice of priors and Markov chain Monte Carlo settings
☒	☐	For hierarchical and complex designs, identification of the appropriate level for tests and full reporting of outcomes
☐	☒	Estimates of effect sizes (e.g. Cohen's d, Pearson's r), indicating how they were calculated

Our web collection on statistics for biologists contains articles on many of the points above.

Software and code

Policy information about availability of computer code

Data collection	BD FACSDiva™ V6 was used to collect flow cytometry data and Gen 5 v3.0.2 was used to collect ELISA data. Mass spectrometry data were collected using MaxQuant version 1.6.3.3. See methods section for more details.
Data analysis	Flowjo V10.6.1 was used to analyze flow cytometry data. GraphPad Prism V9.1.2 was used to analyze and draw corresponding graphs. Stata V15.1 was used to derive threshold values, test regression models and draw survival estimate graphs. Peptides from mass spectrometry experiements were quantified using a label free method based on the MaxLFQ algorithm, analyzed in R v3.6.1 and the Perseus software package version 1.6.6.0. See methods section for a detailed description.

For manuscripts utilizing custom algorithms or software that are central to the research but not yet described in published literature, software must be made available to editors and reviewers. We strongly encourage code deposition in a community repository (e.g. GitHub). See the Nature Portfolio guidelines for submitting code & software for further information.

Data

Policy information about availability of data

All manuscripts must include a data availability statement. This statement should provide the following information, where applicable:

- Accession codes, unique identifiers, or web links for publicly available datasets
- A description of any restrictions on data availability
- For clinical datasets or third party data, please ensure that the statement adheres to our policy

The study protocol and outcomes are published 15,52. The processed human immune response data generated in this study have been deposited at https://dataverse.harvard.edu/dataset.xhtml?persistentId=doi:10.7910/DVN/VNIM1A in a Source Data file and are available under restricted access because of the small number of volunteers and potential privacy concerns. The raw human immune response data are protected and are not available due to data privacy laws. The

Source Data file will be available to researchers who submit forms specifying a research purpose to a Data Governance Committee (contact details and forms available on https://dataverse.harvard.edu/dataverse/kwtrp)). Response times are between 4-6 weeks. The mass spectrometry proteomics raw data have been deposited in the ProteomeXchange Consortium via the PRIDE [1] partner repository with the dataset identifier PXD033964. The peptides that were identified were searched in the Plasmodium falciparum (https://www.uniprot.org/proteomes/UP000001450) and Homo sapiens (https://www.uniprot.org/proteomes/UP000005640) databases at Uniprot.org under default settings. The processed mass spectrometry data and accompanying R code are open access and available at the KWTRP Harvard Dataverse repository at https://dataverse.harvard.edu/dataset.xhtml?persistentId=doi:10.7910/DVN/VNIM1A.

Field-specific reporting

Please select the one below that is the best fit for your research. If you are not sure, read the appropriate sections before making your selection.

☒ Life sciences ☐ Behavioural & social sciences ☐ Ecological, evolutionary & environmental sciences

For a reference copy of the document with all sections, see nature.com/documents/nr-reporting-summary-flat.pdf

Life sciences study design

All studies must disclose on these points even when the disclosure is negative.

Sample size	The primary objective of the original study was to measure correlations between antibody levels to defined and well-characterized malaria antigens and growth rates of P. falciparum in volunteers undergoing controlled human malaria infection (CHMI, reference 52). A sample size of 200 volunteers was estimated for multivariable modeling using the powerreg facility in STATA V13, assuming an r-squared value of 0.3 for a combined model (i.e. including all variables). This had 80% power to detect a single variable that accounted for 0.15 of the variability in growth rates after adjusting for other variables.<br>Only 161 volunteers completed the challenge study. Of these, 19 were excluded from further analysis because they were either found to have antimalarial drugs in plasma (n = 12) or parasite genotypes other than the NF54 strain utilized in the challenge (n=7). We therefore analyzed samples from all remaining volunteers (n = 142) as reported in reference 15.<br>For the Junju cohort, n = 37 samples from adults living in the Junju sublocation of Kilifi County in Kenya were used for assay development, optimization and as positive controls.
Data exclusions	Nineteen of the CHMI adults were excluded from the analyzed data due to detectable levels anti-malaria drugs in plasma (n=12) or the presence of parasite genotypes other than the NF54 strain used in the challenge study (n=7) as reported in reference 52.
Replication	Technical and/or biological replicates were undertaken for all experiments and details for each are provided with each figure in the results and in the supplementary information. All attempts at replication were successful.
Randomization	Randomization was not relevant to this study as it was designed to test the outcome of a standardized dose of parasite inoculum administered to semi-immune adults who varied in the amount of pre-existing anti-malarial antibody. The volunteers were pre-screened to ensure that we had sufficiently variability in the pre-existing antibody response i.e. individuals with low, medium and high levels of antibodies to P. falciparum parasite schizont extract, as reported in reference 52.
Blinding	The investigators conducting the immunological assays were blinded to the clinical outcome until data collection was completed. They were unblinded at the time of data analysis. The experimental design for mass spectrometry involved the comparative analysis of parasite-infected and non-infected red blood cells exposed to a range of predefined treatments that could not be blinded.

Reporting for specific materials, systems and methods

We require information from authors about some types of materials, experimental systems and methods used in many studies. Here, indicate whether each material, system or method listed is relevant to your study. If you are not sure if a list item applies to your research, read the appropriate section before selecting a response.

Materials & experimental systems

n/a	Involved in the study	
☐	☒	Antibodies
☐	☒	Eukaryotic cell lines
☒	☐	Palaeontology and archaeology
☒	☐	Animals and other organisms
☐	☒	Human research participants
☐	☒	Clinical data
☒	☐	Dual use research of concern

Methods

n/a	Involved in the study	
☒	☐	ChIP-seq
☐	☒	Flow cytometry
☒	☐	MRI-based neuroimaging

Antibodies

Antibodies used	Immunofluorescence assays (IFAs)<br>Alexa Fluor® 647 mouse anti-human IgG Fc Antibody, Biolegend UK Limited, catalogue no. 410714, clone M1310G05, lot no. not

available, dilution 1/500.
FITC mouse anti-human CD235a (Glycophorin A) Antibody, Biolegend UK Limited, catalogue no. 349103, clone HI264, lot no. B206676, dilution 1/500

Flow cytometry
APC anti-human CD235a (Glycophorin A) Antibody, catalogue no. 409306, clone HP6017, lot no. B232398, dilution 1/50

Total IgG ELISA
Polyclonal rabbit anti-human IgG, specific for gamma chains, HRP antibody, Agilent (Dako), California, USA, catalogue code P0214, clone DK25, lot no. 20043833, dilution 1/2500

IgG subclass ELISA
Sheep anti-human IgG1, The Binding Site, GmBH, Germany, Ref. AP006, Lot number 426854-1, dilution 1/1000
Sheep anti-human IgG2, The Binding Site, GmBH, Germany, Ref. AP007, Lot number 426686-1, dilution 1/1000
Sheep anti-human IgG3, The Binding Site, GmBH, Germany, Ref AP008, Lot number 426692-1, dilution 1/1000
Sheep anti-human IgG4, The Binding Site, GmBH, Germany, Ref. AP009, Lot number 426695-1, diluted 1/1000
Clone numbers are not available for the subclass antibodies

Validation

Immunofluorescence assays
As explained in part in the results section, we validated our antibodies using a range of experimental controls described below. Parasite invasion of erythrocytes is mediated through multiple receptors including Glycophorin A (CD235a). This is a type 1 sialoglycoprotein present in the cell membrane of erythrocytes and erythrocyte precursors. We therefore used a Fluorescein Isothiocyanate (FITC) anti-human CD235a (Glycophorin A) antibody (Biolegend UK Limited) in a 1:500 dilution as a positive control to localize antibody binding to the surface of infected and non-infected erythrocytes. To detect human antibodies binding to parasite-derived proteins deposited on the surface of erythrocytes, we used Alexa Fluor® 647 anti-human IgG Fc antibodies (Biolegend UK Limited) at a 1:500 dilution for secondary staining in IFAs with malaria-immune plasma at a 1:1000 dilution. Since human antibodies could potentially bind to both Glycophorin A as well as parasite derived antigens, we included two negative controls. The first was malaria-naive plasma to exclude antibody detection potentially arising only from the detection of Glycophorin A. The second used only the Alexa Fluor secondary antibody (without malaria-immune plasma) to exclude non-specific antibody binding. Mature stage parasites express variant surface antigens on the surface of infected parasites that are known to be antibody targets of protective immunity against malaria. We compared binding to rIEs (our hypothesis) to that observed against erythrocytes infected with mature stage parasites (mIEs) as an additional positive control.

Flow cytometry
The validation of the antibodies used in flow cytometry was similarly conducted using positive and negative controls as described for the IFAs above.

ELISAs
Standard titration experiments were conducted with total IgG and IgG subclass antibodies to identify the minimal concentration that allowed the optimal discrimination of antibody detection between malaria-immune and malaria-naive plasma.

Eukaryotic cell lines

Policy information about cell lines

Cell line source(s)	THP 1 cell line - KWTRP
Authentication	Cell viability was checked using Trypan blue staining and counting done using a haemocytometer. Cell growth was maintained at between 1 x 10^5 to 1 x 10^6 by passaging every 5 to 6 days. The number of passages were monitored and kept below 10 as the cells have been reported to be less responsive above this. Our experimental setup included malaria-immune and malaria-naive samples as positive and negative controls. We detected significantly different levels of phagocytosis between control samples that gave us the confidence to quantify phagocytosis levels in individual samples from the CHMI study.
Mycoplasma contamination	The cell lines were not routinely checked for mycoplasma contamination
Commonly misidentified lines (See ICLAC register)	We did not use any commonly misidentified cell lines

Human research participants

Policy information about studies involving human research participants

Population characteristics	Healthy volunteers were recruited for a malaria challenge study. They were aged between 18 ad 45, females and males were equally eligible. We excluded participants with sickle cell trait as this is known to confer protection against malaria. We excluded volunteers with major infections including HIV, Hepatitis B and C or those with clinical malaria. Full details are published in reference 52. For the Junju adults, healthy volunteers were invited to participate in sero-epidemiological studies of malaria immunity. Details provided in the methods section.
Recruitment	The primary objective of the original study was to measure correlations between antibody levels to defined and well-characterized malaria antigens and growth rates of P. falciparum in volunteers undergoing controlled human malaria infection (CHMI, reference 52). To capture the full diversity of antibody responses against malaria parasites, we recruited volunteers from three regions with variable malaria transmission intensities. These were Ahero (high transmission), Kilifi South (moderate transmission) and Kilifi North (low transmission). Healthy individuals aged 18 to 45 were invited to participate. Of the n = 504 that were assessed for eligibility, 185 were excluded for a variety of reasons including major infections and pregnancy. A further n = 149 were excluded ahead of CHMI for additional reasons such as detectable malaria infections or the lack of effective contraception. Study volunteers were required to take up residence in a local university guest house for 24 days. This, together with the requirement for effective contraception may have limited the participation of women. The samples for the analysis presented here came from 44 women and 98 men. Importantly, sex was not a predictor of our outcome measure and therefore we do not think this introduced significant bias. Geographical regions may have introduced bias but were deliberately selected to capture variation in antibody responses. Any potential confounding arising from this was adjusted for in the analysis.
Ethics oversight	For the CHMI study, KEMRI Scientific and Ethics Review Unit (KEMRI//SERU/CGMR-C/029/3190) and the University of Oxford Tropical Research Ethics Committee (OxTREC 2-16). For the Junju adults, ethical approval was provided by the Kenyan National and Scientific Ethics Review Committee protocol number 3149. These details are included in the manuscript.

Note that full information on the approval of the study protocol must also be provided in the manuscript.

Clinical data

Policy information about clinical studies

All manuscripts should comply with the ICMJEguidelines for publication of clinical research and a completedCONSORT checklist must be included with all submissions.

Clinical trial registration	NCT02739763
Study protocol	https://www.ncbi.nlm.nih.gov/pmc/articles/PMC6871356/pdf/wellcomeopenres-3-17028.pdf
Data collection	Volunteers were recruited from areas in Kenya with varying levels of malaria transmission intensity. Ahero ia a town in Kisumu County of Western Kenya. The intensity of malaria transmission is moderate-to-high transmission and the community age–adjusted parasite rates [PfPR] are 40%). In the South of Kilifi County along the Kenyan coast, volunteers were recruited from Junju village, an area of moderate malaria transmission intensity with a current PfPR of 20%, although historically it has been as high as 40%. In the North of Kilifi County, volunteers were recruited from Ngerenya village, an area low-to-no malaria transmission at present but that historicalyy had a PfPR of 25%; see reference 52.
Outcomes	The primary outcomes were pre-specified in the study protocol as clinical symptoms of malaria accompanied by any parasitaemia or parasitaemia exceeding a predefined conservative threshold of 500 parasites/ul of blood. These are described in the manuscript

Flow Cytometry

Plots

Confirm that:

- [x] The axis labels state the marker and fluorochrome used (e.g. CD4-FITC).
- [x] The axis scales are clearly visible. Include numbers along axes only for bottom left plot of group (a 'group' is an analysis of identical markers).
- [x] All plots are contour plots with outliers or pseudocolor plots.
- [x] A numerical value for number of cells or percentage (with statistics) is provided.

Methodology

Sample preparation	All antibodies used were initially titrated and validated using human peripheral mononuclear cells (PBMCs). BD Comp beads were used to adjust for compensation. See methods section for details
Instrument	BD FACS Canto II
Software	BD FACS DIVA V6, Flowjo V10.6.1, R v3.6.1 and Perseus v1.6.6.0
Cell population abundance	Acquired a minimum of 100,000 events

Gating strategy	Dual staining of parasites and THP1 cells was established: the THP-1 cells stained positive for only the CellTrace dye and dual positive for both CellTrace and dihydroehtidium DHE dye respectively when both parasites and THP1 cells were present. See Supplementary Figure 6

☒ Tick this box to confirm that a figure exemplifying the gating strategy is provided in the Supplementary Information.
